# Supplementary material for: Are carbon emissions trading and green financial instruments synergistic? -Comprehensive quantitative research based on content analysis
Source: PLoS One. 2024 Mar 7;19(3):e0298601. doi: 10.1371/journal.pone.0298601 (PMC10919601; doi:10.1371/journal.pone.0298601)
Supplement: S1 Data — (ZIP) [file pone.0298601.s001.zip › Data/地级市Z_F工作报告.zip!/║■▒▒/╗╞╕╘╩╨/└┤╫╘╣1⁄2╓┌║┼í╛┬φ┐╦╩2╛▌═°í┐_2013.docx]

各位代表：

现在，我代表市人民政府向大会报告工作，请予审议，并请市政协各位委员和列席人员提出意见。

一、2012年工作回顾

刚刚过去的2012年，是全市人民攻坚克难、砥砺奋进的一年。面对日趋严峻的宏观经济环境，我们在省委、省政府和市委的坚强领导下，深入贯彻落实科学发展观，大力实施开创战略，着力稳增长、调结构、抓改革、惠民生，锐意进取，扎实工作，较好地完成了市四届人大一次会议确定的目标任务。

——经济发展再上新水平。预计全年实现地区生产总值1190亿元，增长11.3%；规模以上工业增加值326亿元，增长17%；全口径财政收入153.9亿元，增长14.6%，其中公共财政预算收入62.7亿元，增长21.6%；地方税收占公共财政预算收入的比重达到64.2%。主要经济指标增幅高于全省平均水平。

——城乡建设实现新突破。6个县市被列为集中连片特困地区，进入全国扶贫开发主战场。黄冈被批准为全国农产品现代流通试点市、全国三网融合试点市、全国资源综合利用示范基地，蝉联全国双拥模范城。所有县市区均列为全国小农水重点县。九资河镇成为全国低碳绿色示范小城镇试点。374个村被确定为全国农村环境连片整治示范村。大别山试验区实现黄冈全域覆盖。黄梅小池被定位为湖北长江经济带开放开发的“一堡三区一城”。黄冈的改革发展再次上升为国家和省级战略。

——民生改善取得新成效。城镇居民人均可支配收入16720元，增长13.5%；农民人均纯收入6254元，增长15%。城乡居民养老保险和基本医疗保险制度实现全覆盖。居民消费价格涨幅为2.9%。主要污染物排放总量连续7年下降。

一年来，我们主要做了以下工作：

（一）抓投资上项目，迎难而上保增长。深入开展“重大项目建设年”活动。全社会固定资产投资首次突破千亿元大关，达到1035亿元，增长25.8%。争取中央和省预算内投资43亿元。争取大别山试验区产业发展基金12.7亿元。亿元以上在建施工项目290个，增长75.8%。大别山试验区60个“点中点”规划项目647个，完成投资473.8亿元。完成交通投资70亿元，居全省第三位。市区昆仑能源和中粮产业园、团风华中闽台产业城、红安百亿产业群、麻城管材产业群、罗田大别山百里生态画廊、英山茶产业城、浠水陶瓷产业园、蕲春李时珍医药园区、武穴火车站工业园、黄梅纺织服装产业园、龙感湖循环经济产业园等重大产业建设项目全力推进。黄冈长江大桥、九江长江公路二桥胜利合龙，武汉至黄冈城际铁路黄冈段全线贯通，麻武高速、黄鄂高速、麻竹高速、黄冈大道等重大基础设施项目全面启动，大别山红色旅游公路支线建设和配套服务体系建设快速推进，武穴长江大桥、罗霍洲大桥、黄冈机场等一批重大项目前期工作进展顺利。提高招商引资质效。坚持大招大引、招大引大，新引进外来投资项目615个,到位资金248.5亿元。招商引资到位资金、单项规模和建设速度刷新历史记录。大力启动民间投资。放宽领域，降低门槛，积极开展“市场主体增量”行动，个体工商户发展到24.3万户，私营企业达到1.6万户，分别增长18.5%和14.8%。民间投资占全社会固定资产投资总额的比重达到72%，增幅为32.7%。

（二）抓调整转方式，协同带动增实力。全力推进新型工业化。一手抓壮大规模，一手抓调整结构，推动生产要素向优势产业聚集，引导工业项目向园区集中。规模以上工业企业882家，净增121家。产值过亿元企业257家，新增51家。建立完善企业上市“绿色通道”，培育省级后备企业23家，启动中小企业集合债券前期工作。湖北天丰科技在上海股权托管交易中心成功挂牌。荣膺“中国窑炉之乡”、“中国钢结构产业基地”。黄冈经济开发区向国家级开发区迈进。全力推进现代农业。农民收入实现“六连快”，粮食生产实现“九连增”，主要农产品产量全面增长。百亿奶牛产业规划通过国家评审。农产品加工业产值突破440亿元，规模以上农业龙头企业达到204家。农村土地流转面积扩大到47.8万亩，农民专业合作社发展到2589家，地理标志保护产品系列产值41.5亿元，水产品出口创汇1029万美元。加强农业基础设施建设，争取各类项目资金50.8亿元。小（1）型水库除险加固工程全部完工，小（2）型水库除险加固工程开工245座，实施中小河流治理项目8个。农业机械化水平有所提高。全力推进现代服务业发展。推出“多情大别山、风流看黄冈”全新旅游品牌形象，启动大别山号旅游专列，新增4A级景区2家。全年接待游客1362万人次，增长36%；旅游总收入75亿元，增长37%。深入推进“万村千乡”、“农超对接”和区域批发市场、城区集贸市场建设，完成农家直营店建设70家，农家店信息化改造950家。实现社会消费品零售总额547亿元，增长15.6%。全力推进高新技术产业发展。设立省级工程技术中心、研发中心、院士工作站20家，建成省级高新技术产业园2个、高新技术企业32家，完成高新技术产业增加值71亿元。全面落实质量发展纲要，新增湖北名牌产品32项，企业标准覆盖率达到96.6%。

（三）抓市区带县市，统筹发展上水平。高昂市区龙头。坚持“六个统一”，深入推进市区一体开发。市区工业园区规划面积扩大到70.2平方公里，引进亿元以上工业项目45个，协议投资443亿元。大力推进城市建设，完成货币工程量34亿元。城东新区道路、桥梁、白潭湖治理工程和社会事业项目进展顺利，遗爱湖公园8个景区建成开放，市区主次干道全面刷黑，旧城改造、城中村改造、园林绿化全面提升。推动“四城联创”，实施“城管会战”，城市管理水平显著提高。加快县域经济发展。加强分类指导，充分调动各地发展的积极性。5个县市区规模以上工业企业过百家，3个县市固定资产投资过百亿，6个县市财政收入过十亿。全面推进县市城区和小城镇建设，全市城镇建成区面积扩大到190.8平方公里，城镇化率提高1.7个百分点。加强新农村建设。村庄建设完成投资12.2亿元，建设宜居村庄示范项目107个。农村环境连片整治示范工程完成投资2.9亿元，建成生态文明村253个。“难点村”治理全部通过国家验收。大别山红色旅游公路沿线建设秩序和石材行业综合整治工作扎实推进，乱建乱采现象得到有效遏制。农村生产生活条件不断改善，新建高产农田50万亩，新增通村公路883公里，整治塘堰4.5万口，完成清洁能源入户6万户，新增农村安全饮水人口18.7万人。全省最大的农村安全饮水工程在浠水县启动。全国农村公路建设现场会在我市召开。

（四）抓改革促开放，创新驱动添活力。创新投融资机制。金融机构新增贷款首次突破百亿元，大别山地区首家外资金融机构落户麻城。市城投公司10亿元市政项目建设债券成功发行。落实林权抵押贷款1.1亿元。商业保险业务保持高速增长。创新土地利用机制。全力推进城乡建设用地增减挂钩、低丘缓坡土地综合开发利用两项试点，完成拆旧复垦3.3万亩，平整低丘缓坡1.5万亩，落实可用土地指标3.7万亩，城乡建设用地需求得到有效保障。创新跨江跨区合作机制。黄梅小池开放开发融入鄂赣两省战略，总体规划编制完成，湖北大道、清江大道等重点项目建设快速推进，协议引资超过250亿元。浠水与黄石跨江联合开发正式启动。创新科技人才支撑机制。引进“两院”院士及其团队30多人、科技副职和博士服务团70多人，面向全国招考急需人才近3000人，其中硕士研究生以上学历500人。创新扶贫开发机制。整村推进148个村，扶贫搬迁1309户，“雨露计划”培训1.6万人，9.6万贫困人口脱贫。大力推进各项改革。事业单位岗位设置管理全面铺开，其他事业单位绩效工资制度改革正式启动。医改三年行动计划确定的五项重点工作顺利完成，城乡基本医疗保险参保人数达到729.7万人，公立医院改革试点患者付费下降9%，国家基本药物制度实现全覆盖。营业税改征增值税试点工作有序进行。文化体制改革和邮政监管体制改革基本完成。国企改革进一步深化，国有资产监管得到加强。扩大对内对外开放。加大外资、外贸、外经工作力度，推进同央企、知名民营企业战略合作。实际利用外资3200万美元，增长38.6%；外贸出口3.2亿美元，增长25.2%。

（五）抓实事强服务，改善民生增幸福。坚持教育优先发展。全面完成校舍安全工程建设，基本消除D级危房。新建思源实验学校9所、乡镇公办幼儿园116所，建成义务教育标准化学校200所。市区中小学布局调整稳步推进。4所中职学校列入国家发展改革示范学校，黄冈职院开展国际合作办学，黄冈师院向多学科综合性大学迈进。加强文化建设。完善公共文化服务体系，乡镇综合文化站、标准农家书屋实现全覆盖。黄冈博物馆建成开放。启动实施名人文化建设和文物事业发展规划，特色文化产业加快发展。《东坡》、《李时珍》等戏曲精品登上国家级舞台，电影《全城高考》被推荐为优秀国产影片。荣获首届湖北省艺术节奖项21个。成功举办了第三届东坡文化节、第二届挺进大别山自行车赛和首届全国汽车场地越野锦标赛。提高社会保障水平。全市参加五项社会保险人数达到296万人，医疗、工伤、生育保险实现市级统筹，社会保险关系实现无障碍转移接续，“金保工程”上线运行，社会保障卡全面发行。城镇新增就业7.1万人，帮助农村富余劳动力转移就业7.8万人，城镇登记失业率3.9%。积极推进“健康黄冈”全民行动。新农合专网连接所有村卫生室，在全省率先建成新农合市级转诊信息平台。转变人口计生工作方法，推行“看表管理”，强化综合控比，深化优质服务，全面完成省定责任目标。着力完善社会救助体系，取消大病救助病种限制，加强农村福利院建设，城乡低保对象53.7万人，发放低保及救助资金9.6亿元。深入推进六大食品安全工程和放心早餐工程。保障性住房开工18186套、竣工13366套，改造农村危房26550户、竣工20176户。市政府承诺的10件实事全面完成。

（六）抓管理提效能，依法行政促和谐。大力推进法治黄冈建设，主动接受市人大、市政协的法律监督、工作监督和民主监督，办理省市两级建议提案547件，代表、委员满意和基本满意率都达到100%。建议提案办理工作受到省人大、省政府、省政协联合表彰。“六五”普法扎实开展。加强国防动员、民兵预备役建设，做好“双拥”工作，积极开展军民共建活动。退役士兵安置工作经验在全国推广。密切联系工会、共青团、妇联等群众团体，广泛听取社会各界意见和建议。着力推进以社会管理“三化三全”为龙头的政法工作“六大工程”，进一步加强社会管理综合治理，深入排查化解社会矛盾，妥善处理各类突发事件，积极构建社会治安防控体系，严厉打击违法犯罪，社会治安保持稳定，群众安全感不断增强。安全生产态势保持平稳，应急管理能力进一步增强。深入开展群众性精神文明创建活动，涌现出一批英雄模范人物。着力打造最优发展环境。市级行政审批事项由477项精减至168项，收费项目由109项减少到13项，审批环节、办理时限、申报资料分别下降60%、76%、46%。加大政府信息公开力度，创新政务公开方式，推行电子政务，市县乡村四级政务服务体系进一步完善。强化审计、监察、行政复议工作，深入开展以工程建设领域为重点的源头治理，大力纠正损害群众利益的不正之风，廉政建设不断加强。招投标综合监管体系基本形成。机关事务管理走上规范化轨道。对口援建任务超额完成。妇女儿童发展规划全面实施。民族、宗教、人防、消防、地震、仲裁、统计、调查、保密、档案、方志等方面的工作，都取得了新的进展。

过去一年，我市经济社会发展取得的成绩，得益于省委、省政府和市委正确领导，得益于全市人民团结奋斗，得益于社会各界大力支持。在此，我谨代表市人民政府，向辛勤工作在各行各业的全体市民，向给予政府工作大力支持的人大代表和政协委员，向各民主党派、工商联和社会各界人士，向驻黄部队和武警官兵，向所有关心支持黄冈改革发展的同志们、朋友们，表示崇高的敬意和衷心的感谢！

回顾过去一年的工作，我们也清醒地认识到，政府工作与上级的要求和全市人民的期待还有差距，经济社会发展中仍存在不少突出矛盾和困难，主要是：经济规模不大，产业竞争力不强，发展水平较低，当年地区生产总值、规模以上工业增加值等指标增幅未完成市四届人大一次会议确定的目标；城市建设相对滞后，城镇化水平较低，社会事业欠账较多；城乡居民收入水平不高，部分群众生活困难，就业、社保、医疗、住房、教育等民生问题仍然突出，公共服务水平还需进一步提高；行政效能和服务水平还有差距，公务员队伍能力和素质有待进一步提高，发展环境还需进一步优化。对此，我们将进一步增强忧患意识和责任意识，采取有效措施，认真加以解决。

二、2013年主要任务

今年是全面贯彻落实党的十八大精神的开局之年，是实施“十二五”规划承前启后的关键一年，是为全面建成小康社会奠定坚实基础的重要一年。今年政府工作的总体要求是：深入学习和全面贯彻落实党的十八大和中央、省经济工作会议精神，坚持以科学发展观为指导，围绕主题主线、突出质量效益、深化改革开放、强化创新驱动，着力调整优化经济结构，着力推动“四化”同步发展，着力保障和改善民生，确保黄冈大别山试验区建设实现明显变化，奋力推进全市经济社会科学发展、跨越发展。

今年全市经济社会发展的主要预期目标是：地区生产总值增长12%左右；全社会固定资产投资增长25%以上；规模以上工业增加值增长18%以上；社会消费品零售总额增长16%以上；外贸出口和实际利用外商投资增长15%以上；公共财政预算收入增长16%以上；城乡居民可支配收入增长12%以上；居民消费价格涨幅控制在4%以内；城镇登记失业率控制在4.6%以内；节能减排完成省定任务；人口自然增长率控制在6‰以内。

确定上述目标，既着眼于全面建成小康社会的要求，又注重了与“十二五”规划相衔接，充分考虑了需要与可能，有利于引导各方面把主要精力放到加快转变经济发展方式、提高经济增长质量和效益上来。黄冈要实现后发赶超，必须紧紧扭住经济建设这个中心，正确处理稳增长与转方式、调结构、增效益的关系，坚持在发展中转变、在发展中调整、在发展中增效，保持较快的发展速度，为扩大就业、增加收入、保障民生提供强大的物质支撑。

今年要重点抓好十个方面工作：（一）着力转变经济发展方式

以扩大有效投入为抓手增强发展后劲。继续把项目建设摆在经济工作首位，更加扎实地做好项目工作，推动投资持续快速增长，确保完成全社会固定资产投资1300亿元。坚持超前谋划、主动作为，着眼于产业升级和城市功能提升，依托资源优势和现有产业基础，精心策划一批先进制造业、现代服务业、现代农业、高新技术产业、城乡基础设施、社会事业和民生工程等重大项目。抓紧做好铁路、高速公路、机场、长江大桥、港口码头和电厂等重点项目的前期工作，力争尽早立项动工。健全重点项目推进机制，明确各地各部门项目工作责任，继续实行一个项目一个专班，跟踪协调，全程服务。重点抓好400个投资过亿元的项目建设，继续抓好昆仑能源、东坡外滩、麻武高速、武穴长江大桥等200个重点续建项目，确保黄冈长江大桥、武汉至黄冈城际铁路建成通车。

以培育新的增长点为目标扩大消费需求。着力增加城乡居民尤其是中低收入者收入，提高居民消费能力。拓宽消费领域，促进文化、旅游、教育、健身等消费，培育一批拉动力强的消费热点。整顿市场秩序，畅通维权渠道，规范价格和收费管理，进一步降低流通成本，继续实施食品药品安全工程、放心早餐工程、放心粮油配送工程，有效应对交通流通格局变化，着力打造区域最优消费环境。编制现代服务业和商业网点建设规划，有序推进商业街建设，新建和改造一批商场、批发市场、农贸市场等商品流通设施。支持流通企业向农村延伸商业网点，提升农民专业合作社物流配送能力和营销水平，畅通农产品进城和工业品下乡双向流通渠道。扎实推进黄州现代服务业综合试点建设。深入开展中国优秀旅游目的地创建活动，培育旅游市场主体，做大旅游投融资平台，整合资源资产，扩大市场营销，完成大别山红色旅游公路服务体系建设，推动旅游业更好更快发展。加快金融保险、信息服务、科技咨询、服务外包、房地产等服务业健康快速发展。

以科技创新为手段提升产业竞争力。加快实施一批科技专项，推进高新技术产业化。加快以黄冈高新区为龙头的高新技术产业园区建设，促进高新技术产业集聚发展。加强知识产权保护。完善创新创业中介服务体系，加快生产力促进中心和孵化器建设。进一步完善科技合作机制，组建战略联盟，搭建产学研合作平台。创新科技政策支持机制、科技金融结合机制、科技人才工作机制，营造良好科技工作环境。

（二）着力推进新型工业化

打造大园区。把工业园区作为推进新型工业化的有效载体和主要阵地，创新体制机制，拓宽投入渠道，提升硬件功能，强化配套服务，拓展发展空间。力争黄冈经济开发区创建国家级开发区。抓紧规划建设中关村·黄冈产业园、中国光谷·黄冈产业园、黄冈光热产业园、融园食品产业城和中船重工军民结合产业园。采取有效政策措施，集中各种经济要素，加快推进省级开发区建设和发展。支持县市区集中连片建成10平方公里以上的工业新城，力争工业园区基础设施建成区实现项目满园，2—3个园区规模以上工业产值突破百亿元。

建设大项目。把招商引资作为项目建设的第一抓手，加强规划招商、以商引商、产业链招商，引进和建设一批科技含量高、经济效益好、产业带动力强的项目。大力支持昆仑能源日产500万方液化天然气、湖北华海大型船舶专业化生产、黄冈伊利HDPE瓶装果汁酸奶生产线、湖北迅达药业原料药生产基地、湖北燕加隆年产200万方环保地板、湖北五瑞年产1.5亿单位低分子肝素钠等37个产业振兴项目建设。把支持重点骨干企业做大做强作为生成大项目的重要途径，引导企业加强技术改造和信息化建设。力争完成工业投入550亿元，增长25%以上。把引进培育大项目与推进中小企业成长工程结合起来，完善“小进规”奖励机制，确保全年新增120家规模以上工业企业。

发展大产业。巩固提升食品饮料、医药化工、机械电子、纺织服装、建筑建材等产业，加快发展造船、钢构、窑炉、陶瓷等产业，合力打造火电、水电、风电、抽水蓄能发电、生物质能发电等能源产业，大力培育节能环保、新型材料、电子信息、生物工程等战略性新兴产业。加强大别山试验区产业发展基金管理、使用与监督，发挥产业基金引导激励作用。深入开展“品牌建设年”活动，加强质量标准和品牌建设，推动产业向“价值微笑曲线”两端延伸，打造黄冈工业的标志和品牌。

优化大环境。牢固树立“产业第一、发展优先”的理念，建立健全领导挂点联系服务企业制度，加强企业“直通车”服务工作，健全中小企业社会化服务体系，培育一批服务示范机构，打造一批特色服务品牌。高度重视企业家队伍建设，鼓励创业、尊崇创新、支持创富，切实让企业家人才辈出、活力迸发。

（三）着力推进农业现代化

提高农业市场化水平。围绕农业大市向农业强市转变、传统农业向现代农业转变，用工业理念谋划农业，用现代管理经营农业，用农产品加工引领农业，用品牌战略打造农业。大力引进和培育一批农产品加工龙头企业，建设一批农产品加工园区和农产品加工大县。着力打造粮食、油料、奶牛、纺织、地标产品、畜禽、水产、园艺、中药材、森工10个百亿产业，加快形成现代农业千亿产业链。继续开展省级示范合作社创建。完善农企利益联结机制，推广土地入股和龙头企业带动模式。

加强农村基础设施建设。继续推进水库除险加固、中小河流治理、山洪灾害防治、灌区续建配套与节水改造、生态修复治理、农村饮水安全等工程建设。高标准实施国家小农水重点县建设，扩大旱能浇、涝能排高产农田面积。加强农村交通安保工程建设，切实提高通村公路管理养护水平。加强地质灾害防治，推进农业气象服务体系和农村气象灾害防御体系建设。落实防汛抗旱责任制，努力把各种灾害损失降到最低程度。

完善农业科技创新体系。加快构建和完善农业社会化服务体系，重点解决农技推广“最后一公里”问题。抓好科技推广阵地和科技示范户建设，提高农民的科学种养技能和经营水平。大力推行农产品质量安全标准和生产技术规程，加快建设农业标准化示范区和生产基地。充分挖掘国家地理标志保护产品的品牌优势和市场潜力。加快农业机械化步伐。

多渠道增加农民收入。认真落实强农惠农富农政策，增加农民转移性收入。扩大农民转移就业规模，增加农民工资性收入。完善农村产权制度和征地制度，积极稳妥地促进土地承包经营权流转，增加农民经营性收入。力争农民收入增幅超过城镇居民、高于全省平均水平。持续抓好农民负担监管，更好地维护农民合法权益。

全面推进扶贫开发。坚持开发式扶贫方针，实现片区区域发展和扶贫进村入户同步推进，扶贫开发和农村最低生活保障制度有效衔接，专项扶贫、行业扶贫、社会扶贫三位一体，解决8万贫困人口的脱贫问题。

（四）着力推进城镇化

加大市区建设力度。按照“改造老城、建设新城、提升全城”的思路，大力推进“一区十大项目”建设。“一区”，就是加快建设城东新区，重点推进市政务中心、还建小区、道路管网、学校和医疗中心等工程建设。“十大项目”，即遗爱湖公园、赤壁公园、市政道路、城铁站和火车站广场、污水处理厂扩建、自来水厂扩建、旧城改造、背街小巷建设、保障性安居工程和社会事业发展项目。加大城区排污管网建设力度，加强供排水、燃气、市政安全管理，抓好临港新城等港口码头建设。坚定不移地推进市区一体开发，突出抓好园区建设、项目建设和环境建设，实现园区基础设施建设重大突破，新引进亿元以上项目30个，10亿元以上投产项目5个。

加快县域城镇发展步伐。把县市城区和重点镇作为推进城镇化的战略重点，强化经营城镇理念，多渠道筹措建设资金，加快基础设施建设，做大做强特色产业和园区，增强产业发展、公共服务、吸纳就业、人口聚集功能。积极推进城乡一体化试点工作。把发展县域经济作为强市之基，大力提升县域工业化、城镇化水平，争取各县市区在全省考核排名全面提升。推进重点镇特色镇示范工程，实施扩权强镇。有序推进农业转移人口市民化。坚持“省级战略、市县实施”，加快小池开放开发，城镇发展实现“一年一个样”。加大跨江跨区联合开发力度，支持散花等口子镇快速发展。

加强城镇规划和管理。大力推进城镇和村庄规划全覆盖，建立重大建设规划方案竞选机制，提高城镇规划水平。严格执行城乡建设规划，扩大城中村试点范围，坚决制止违法建设。重视历史文化资源和自然风貌保护，提升城市品位。支持公交优先发展。切实解决城市停车难等问题。深入开展“城管会战”，推动实施“数字城管”，建立和完善城乡管理长效机制。大力推进市区新一轮“四城联创”。

（五）着力推进信息化

建设智慧城市。把建设智慧城市作为推动黄冈跨越发展的重要抓手，加快建设以数字化、网络化、智能化为主要特征的智慧城市，全面构建以智慧政务、智慧产业、智慧民生为重要内容的城市发展新模式。加快建设光纤接入网、大容量承载网、下一代互联网和广播电视网。大力推进公共场所无线局域网建设。深化三网融合，创新运营管理模式，支持融合型业务发展。

加快推进企业信息化。突出主导产业和重点企业，大力推进信息技术在产品研发设计、生产过程、企业管理、市场营销、人力资源开发、新型业态培育、企业技术改造等关键环节的应用和推广。建成中小企业信息服务平台。力争全市规模以上企业信息技术应用率达到50%以上，培育市级信息化试点企业30家。

积极发展电子商务。编制全市电子商务发展规划，引导推动营销网、物流网、信息网有机融合。普及和深化电子商务应用，建立认证、支付等支撑体系，鼓励企业建成或依托第三方电子商务平台开展网上交易。推进流通领域公共信息服务平台建设，提升各类信息资源的利用效率。

加快建设“数字黄冈”。扎实推进全国数字城市建设试点工作，完善基础地理空间数据库，实现数据适时更新和“一张图”管理。建立长效机制，推动地理信息技术成果应用。积极推进县级数字城市建设。

（六）着力深化改革开放

扎实推进大别山试验区和“两型社会”综改区建设。全面实施两大试验区总体规划和专项规划，推动落实市厅共建协议，积极争取各类建设项目，继续抓好“点中点”建设。着力深化市场融资、环境保护与生态补偿、循环经济与资源综合利用、城乡一体化等关键领域和关键环节的改革。坚持主动作为、干中求助、全力推动，积极争取大别山革命老区振兴发展上升为国家战略，配合编制起草振兴发展规划及支持政策。

深化经济体制改革。按照国家和省改革路线图和时间表，不失时机深化各项改革，增强经济社会发展的内生动力和活力。深化财税体制改革，推进“营改增”试点工作，加强税收征管，完善部门预算，确保财政收支平衡。深化金融体制改革，完善金融组织体系，加快多层次资本市场体系建设，支持实体经济发展。加快投融资体制改革，规范投融资平台建设，扩大融资规模。探索创新农业生产经营体制机制，培育新型经营主体。进一步推进市直部门企业改革，加强国有资产监管。

大力发展开放型经济。实施更加积极主动的开放先导战略，全方位扩大对内对外开放。精心培育外贸经营主体，加强生物医药、船舶、纺织服装、农产品以及窑炉、茶叶等出口基地建设，不断扩大出口份额。启动联合联检大楼建设，优化通关环境。加强与重点国家和地区、大型跨国公司的战略合作，扩大实际利用外商投资和国外贷款规模。引导企业积极稳妥“走出去”，有重点、有步骤地推动境外资源开发、优势产能转移、对外工程承包。

加快发展民营经济。进一步放宽经营条件，拓宽投资领域，促进民营经济跨越式发展。健全民营企业服务体系，培育一批有潜力的中小民营企业，扶持壮大科技型民营企业，鼓励推动外出成功人士回乡创业。积极推进全民创业，完善落实减负惠企政策措施，促进更多民间资金转化为产业资本，繁荣民营经济。

（七）着力建设美丽黄冈

加强环境保护和生态建设。严格落实节能减排目标责任制，大力实施重点节能减排工程，确保完成省定各项目标任务。坚持调结构、控增量、减存量、提质量，严格审查涉及重金属排放、“两高一资”和产能过剩的行业项目，加强污水和垃圾处理设施建设，加快重点企业环保设施升级改造。稳步推进低碳试点。全面实施清洁水源、清洁空气、清洁土壤三大行动，继续推行河湖库长制，切实解决损害群众健康的环境问题。推进生态殡葬。坚持生态立市，广泛开展植树造林，加强自然保护区、森林公园和湿地公园建设，抓好森林防火和病虫害防控。积极开展森林城市、森林小镇、绿色村庄创建活动。

加强城乡环境综合整治。积极创建省级环保模范城市，实施生态修复工程，切实改善环境质量。加快黄冈长江生态文明示范带建设，扩大农村环境连片整治示范试点，力争110个村进入全国全省整治范围。积极开展生态县、生态乡镇和生态村创建活动。做好大别山世界地质公园申报工作。

加强资源集约节约利用。继续举全市之力推进城乡建设用地增减挂钩试点，抓好低丘缓坡地和闲置低效地综合开发利用，促进土地资源集约节约利用，保障重点项目用地需求。规范矿产勘查开发秩序，继续开展石材行业综合整治。大力推进全国资源综合利用“双百”工程示范基地和绿色能源示范县建设，促进企业循环式生产、产业循环式组合、园区循环式发展。

（八）着力推进文化强市建设

完善公共文化服务体系。推进公共文化服务均衡发展，创建国家级公共文化服务体系示范区。黄冈博物馆规范运行、全面开放。加快黄梅戏剧院、黄冈艺术学校建设步伐，启动市图书馆新馆、市群众文化活动中心建设。完善县市区图书馆、文化馆和乡镇综合文化站，建设一批街道文化中心和社区文化活动室，规范文化信息资源共享工程和农家书屋的管理使用。扶持各类群众文化团体，开展形式多样的文化惠民活动。办好第八届湖北省黄梅戏艺术节。加强重点文物和非物质文化遗产保护，传承城市文脉，丰富文化底蕴。

大力发展文化产业。编制黄冈市文化产业发展战略规划和专项规划，重点打造红色文化、东坡文化、戏曲文化、禅宗文化、医药文化等文化品牌，建设一批国家级、省级文化产业示范基地。加大文化产业招商引资力度，积极构建黄冈文化创意产业园区，着力培育一批骨干文化企业，推出一批可消费、可带走、可持续发展的文化产品。重视生态文化旅游，推动文旅深度融合。

加快文化体制改革创新。实施文化企业公司制股份制改造，建立面向市场的经营机制和体现文化企业特点的管理模式。加快文化事业单位分类改革。探索公共文化设施和项目委托管理，创新社会化、专业化管理运行机制。积极培育和引进优秀文化人才，造就一支高素质文化队伍。加强文化市场监管，营造健康有序的文化市场环境。

（九）着力加强以改善民生为重点的社会建设

努力办好人民满意的教育。坚持教育优先发展，继续加大财政投入，推动教育资源向农村薄弱学校和困难群体倾斜。办好学前教育，实现适龄儿童接受学前教育和看护服务全覆盖。合理调整中小学布局，大力改善办学条件，建立健全教师补充交流机制，推动义务教育均衡发展。优化各级各类学校办学环境，支持黄冈中学做实做强品牌，支持黄冈师院等高等学校提升内涵式发展水平和服务地方经济发展能力。整合职业教育资源，加快建设黄冈职教园区。重视支持特殊教育、继续教育，鼓励引导社会力量兴办教育。

扎实做好就业和社会保障工作。做好以高校毕业生为重点的青年就业工作和农村转移劳动力、城镇困难人员、退役军人就业工作。加强职业技能培训，鼓励劳动者就地就近就业。重视解决本地企业用工难的问题。全面推行社会保险费“五险一单”统一征缴，不断扩大社会保险覆盖范围。建立完善各类人员的社会保障待遇调整机制，稳步做好养老保险制度衔接转换工作。逐步做实养老保险个人账户，做好城镇职工基本养老保险省级统筹，完善医疗、工伤、生育保险市级统筹，实行失业保险市级统筹。构建和谐劳动关系。加强保障性安居工程建设。健全住房公积金制度。完善社会救助体系，规范城乡低保运行，健全社会福利制度，支持发展慈善事业，做好优抚安置工作。加大农村福利院改造建设力度，实现农村“五保”应保尽保。健全残疾人社会保障和服务体系。保障妇女儿童残疾人合法权益。建立多元化老龄事业投入机制，大力发展老龄服务事业和产业。

提高人民健康水平。进一步深化医疗卫生体制改革，推进县级公立医院综合改革，巩固完善基本药物制度和基层运行新机制，推进基本公共卫生服务均等化。健全全民医保体系，完善新农合市级统筹，实施城镇居民医保门诊统筹，探索建立职工医疗保险门诊统筹。改革医疗保险付费方式，全面实现省内异地就医即时结算。严格执行生育政策，深入推进“两非”整治，综合治理出生人口性别比，强化计生利益导向和便民惠民服务，稳定低生育水平，提高出生人口素质。广泛开展全民健身运动，不断提高体育竞技水平。加快黄冈市体育中心建设。

加强和创新社会管理。加快基层社会管理和服务体系建设，推广社区网格化服务管理，增强城乡社区服务功能。创新服务管理，促进流动人口有序融入城镇、特殊人群顺利融入社会。完善信访制度，健全人民调解、行政调解、司法调解联动的工作体系，畅通和规范群众诉求表达、利益协调、权益保障渠道。加强应急管理，提高预防和处置突发公共事件能力。深入开展平安黄冈建设，完善立体化社会治安防控体系，加大“打黑除恶”、反抢反盗等严打工作力度，依法防范和惩治违法犯罪活动，切实保障人民生命财产安全。落实安全生产责任制，强化公共安全体系和企业安全生产基础设施，坚决遏制较大以上安全事故。加强消防工作。建成黄冈市防震减灾中心。

增强全民国防观念，加大国防动员工作力度，加强民兵预备役、人民防空和“双拥”工作，推进军民融合式发展。继续做好援疆工作。依法管理宗教事务，巩固民族团结。切实做好小康社会建设进程监测和经济普查调查工作。认真做好气象、对台、侨务外事、保密、档案、方志等各项工作。

继续办好十件实事。1、城镇新增就业4万人，帮助农村富余劳动力转移就业5万人。2、建设保障性住房30万平方米；改造农村危房15000户。3、新建标准化菜篮子基地10万亩；每个县市区新建或改造1个标准化农贸市场。4、解决农村10万人饮水安全问题；建成农民体育健身工程150个。5、全市建成放心粮油经营示范网点200个、食品安全示范店200家；市区建成放心早餐工程示范店70家。6、新建农村连村通组水泥（沥青）公路1000公里。7、为1000名贫困残疾人适配辅助器具，为200名贫困残疾人家庭实施无障碍改造，为300名0—6岁贫困残疾儿童实施抢救性康复，为4000名残疾人机动轮椅车发放燃油补贴；全面实施80岁及以上高龄老人补贴制度。8、建成义务教育阶段标准化学校260所；新建、改扩建乡镇公办幼儿园50所；启动城东新区3所学校建设。9、深入开展“万名干部进万村洁万家”活动，建成垃圾处理场140个、垃圾处理池4万口，购置垃圾转运车4000台。10、为困难群众和农民工办理法律援助案件1400件、法律援助事项14000件。

（十）着力推进政府自身建设

进一步转变工作作风，密切联系群众。认真落实市委关于转变工作作风、密切联系群众的意见及实施细则，各级政府机关和领导干部带头学习贯彻党的十八大精神，带头开展党的群众路线教育实践活动，带头深入基层调查研究，带头直接联系群众，带头艰苦创业，带头整治庸懒散奢，带头改进会风文风，带头遵守廉政准则，带头解决实际问题，带头推动工作落实。要求县乡政府做到的市政府首先做到，要求下级做到的市政府领导班子成员率先垂范，要求别人不做的自己坚决不做。

进一步加强依法行政，切实打造阳光政务。深入推进法治黄冈建设，健全决策机制，提高决策科学化、民主化、法治化水平。自觉接受市人大及其常委会的法律监督、工作监督和市政协的民主监督，完善向人大报告工作、向政协通报工作制度，认真办理人大议案、代表建议、政协提案，虚心听取各民主党派、工商联、无党派人士和人民团体的意见。加强和规范行政执法，推进行政复议和行政仲裁工作，做到严格执法、文明执法、公正执法。深化政务公开，让公共权力、公共资源、公共服务公开透明。

进一步加强勤政廉政建设，提高政府公信力和执行力。完善政府绩效管理，健全抓执行机制，构建闻风而动、雷厉风行的决策、执行、监督、考核、奖惩“落实链条”，务求办实事、见实效，做到言必信、行必果。加快转变政府职能，全力打造全省最优发展环境。坚持能力席位标准，加强政府机关及公务员能力建设。严格执行党风廉政建设责任制，完善惩治和预防腐败体系，加大大案要案查处力度，以反腐倡廉实际成效取信于民。加强效能监察和审计监督，健全权力运行制约和监督机制。加强节约型机关建设，严格控制“三公”经费，千方百计降低行政成本，挤出更多资金为人民群众办实事、办好事。

各位代表！黄冈正处于实施开创战略、推进跨越发展的大好时期。实现全面建成小康社会的宏伟目标，任重道远，使命光荣。让我们紧密团结在以习近平同志为总书记的党中央周围，在市委的正确领导下，解放思想，改革开放，凝聚力量，攻坚克难，为建设富裕文明和谐的新黄冈而努力奋斗！
